# Postoperative acute exacerbation of interstitial pneumonia in pulmonary and non-pulmonary surgery: a retrospective study

**DOI:** 10.1186/s12931-019-1128-5

**Published:** 2019-07-15

**Authors:** Takuto Miyamura, Noriho Sakamoto, Tomoyuki Kakugawa, Daisuke Okuno, Hirokazu Yura, Shota Nakashima, Hiroshi Ishimoto, Takashi Kido, Daisuke Taniguchi, Takuro Miyazaki, Tomoshi Tsuchiya, Shin Tsutsui, Hiroyuki Yamaguchi, Yasushi Obase, Yuji Ishimatsu, Kazuto Ashizawa, Takeshi Nagayasu, Hiroshi Mukae

**Affiliations:** 1 Department of Respiratory Medicine, Nagasaki University Graduate School of Biomedical Sciences, 1-7-1 Sakamoto, Nagasaki, 852-8501 Japan; 2 Department of Surgical Oncology, Nagasaki University Graduate School of Biomedical Sciences, 1-7-1 Sakamoto, Nagasaki, 852-8501 Japan; 3 Department of Nursing, Nagasaki University Graduate School of Biomedical Sciences, 1-7-1 Sakamoto, Nagasaki, 852-8520 Japan; 4 Department of Clinical Oncology, Nagasaki University Graduate School of Biomedical Sciences, 1-7-1 Sakamoto, Nagasaki, 852-8501 Japan

**Keywords:** Acute exacerbation, C-reactive protein, Interstitial pneumonia, Surgery

## Abstract

**Background:**

Acute exacerbation of interstitial pneumonia (AE-IP) is a serious complication of pulmonary surgery in patients with IP. However, little is known about AE-IP after non-pulmonary surgery. The aim of this study was to determine the frequency of AE-IP after non-pulmonary surgery and identify its risk factors.

**Methods:**

One hundred and fifty-one patients with IP who underwent pulmonary surgery and 291 who underwent non-pulmonary surgery were retrospectively investigated.

**Results:**

AE-IP developed in 5 (3.3%) of the 151 patients in the pulmonary surgery group and 4 (1.4%) of the 291 in the non-pulmonary surgery group; the difference was not statistically significant. A logistic regression model showed that serum C-reactive protein (CRP) was a predictor of AE-IP in the non-pulmonary surgery group (odds ratio 1.187, 95% confidence interval 1.073–1.344, *P* = 0.002).

**Conclusions:**

This is the first study to compare the frequency of AE-IP after pulmonary surgery with that after non-pulmonary surgery performed under the same conditions. The results suggest that the frequency of AE-IP after non-pulmonary surgery is similar to that after pulmonary surgery. A high preoperative C-reactive protein level is a potential risk factor for AE-IP after non-pulmonary surgery.

**Electronic supplementary material:**

The online version of this article (10.1186/s12931-019-1128-5) contains supplementary material, which is available to authorized users.

## Background

Acute exacerbation of interstitial pneumonia (AE-IP) is a serious complication of pulmonary surgery and is associated with high mortality [1–5]. Resection of lung cancer has been reported to be one of the risk factors for AE-IP, and acute exacerbation (AE) has been reported to occur after surgery in 9.5–22.5% of patients with IP [6–13]. A simple risk scoring system that includes type of surgical procedure, patient sex, history of exacerbation, preoperative steroid use, the serum Klebs von den Lungen (KL)-6 level, whether or not usual interstitial pneumonia (UIP) is detected on computed tomography (CT), and percentage predicted vital capacity has been developed in Japan for prediction of AE-IP after pulmonary resection in patients with lung cancer [14]. However, only three studies have investigated postoperative AE-IP in patients undergoing non-pulmonary surgery [1, 15–17]. In one of these studies, AE-IP was reported to occur in 7.8% of patients with idiopathic IP, and the only risk factor identified was administration of propofol [16]. In addition, other study reported that AE-IP occurred 6.3% of patients with interstitial lung disease, and only high preoperative C-reactive protein (CRP) level was identified a risk factor [17]. However, there has been no report comparing the frequency of postoperative AE-IP in patients undergoing pulmonary surgery with that in those undergoing non-pulmonary surgery at the same institution. The first aim of this study was to determine the frequency of postoperative AE-IP in patients undergoing non-pulmonary surgery and compare it with that in those undergoing pulmonary surgery at the same institution. The second aim was to identify the risk factors for postoperative AE-IP in patients who have undergone non-pulmonary surgery.

## Methods

### Study population and covariates

The study protocol was approved by the Institutional Review Board at Nagasaki University Hospital (approval number 17112011) and conducted in accordance with the Declaration of Helsinki. Informed consent was not required in view of the retrospective study design and the anonymity of the patient records reviewed, pursuant to the ethical guidelines of the Japanese Ministry of Health, Labor, and Welfare. The study population comprised a cohort of consecutive patients with IP detected by high-resolution CT (HRCT) who underwent surgery under general anesthesia at Nagasaki University Hospital, Nagasaki, Japan, from April 2008 to October 2017. Preoperative chest HRCT images were used to identify IP. Preoperative patient characteristics, laboratory findings, and anesthetic management obtained from the clinical records were compared between patients who experienced AE-IP postoperatively and those who did not.

### Diagnostic criteria for acute exacerbation

Previously published criteria were used to define postoperative AE-IP, i.e., onset within 30 days after surgery, extra-parenchymal cause excluded, and new bilateral ground glass opacity/consolidation on CT not fully explained by cardiac failure or fluid overload [18]. Patients with cardiac failure and fluid overload were excluded by echocardiography and responded to diuretics. Pulmonary infections were evaluated by sputum and blood culture, and other serological and urinary studies for the following pathogens and pathogen components including *Mycoplasma pneumoniae*, cytomegalovirus antigen, β-D glucan, Legionella spp., and *Streptococcus pneumoniae*.

### Evaluation of HRCT findings

The HRCT scans of the chest were interpreted independently and in random order by two pulmonary radiologists (ST, KA) without knowledge of the clinical status of the patients. Divergent observations were resolved by consensus after consultation between the two observers. All the HRCT scans were acquired in the 6 months before surgery. Chest CT images were classified according to the ATS/ERS/JRS/ALAT guidelines [19]. Based on the guidelines, UIP or probable UIP pattern was categorized into a UIP group and indeterminate UIP pattern or alternative diagnostic findings were categorized into a non-UIP pattern. Patients with UIP or probable UIP pattern of unknown cause were diagnosed to have idiopathic pulmonary fibrosis (IPF).

### JACS risk score

The risk of AE-IP after surgery can be defined according to the JACS (Japanese Association for Chest Surgery) risk score [14]. Briefly, this score is derived as follows: 5 × (history of AE) + 4 × (surgical procedures: segmentectomy, lobectomy, bilobectomy, pneumonectomy) + 4 × (UIP appearance on CT scan) + 3 × (male sex) + 3 × (preoperative steroid use) + 2 × (elevated serum sialylated carbohydrate antigen, KL-6 level: over 1000 U) + 1 × (low vital capacity: less than 80%).

### Statistical analysis

The data are presented as the frequency for categorical variables and as the median and interquartile range (IQR) for quantitative variables. Univariate (Wilcoxon rank-sum and chi-squared tests) and logistic regression analyses were performed to identify differences between the pulmonary surgery group and the non-pulmonary surgery group and possible risk factors for AE-IP. Firth penalized logistic regression was used in the analyses of smoking history, neoadjuvant chemotherapy and lung infection (a surgical complication) in the pulmonary surgery group, male sex, corticosteroid therapy, past acute exacerbation, KL-6 level ≥ 1000 U/mL, and lung infection (a surgical complication) in the non-pulmonary surgery group because of quasi-complete separation. All *P*-values were two-sided and considered statistically significant when less than 0.05. All the statistical analyses were performed using the JMP Pro software program (version 13.0.0; SAS Institute, Inc., Cary, NC, USA).

## Results

### Subject characteristics

**Fig. 1:**
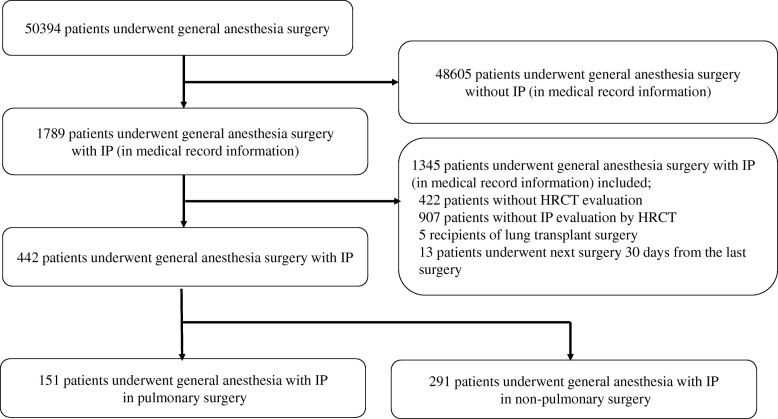
Study design. A retrospective review of clinical records identified 50,394 patients who underwent surgery under general anesthesia from April 2008 to October 2017. Of these, 1789 patients had interstitial pneumonia (IP). In total, 1345 patients were excluded because they had not undergone HRCT (*n* = 422), did not have IP confirmed on HRCT of the chest (*n* = 907), were lung transplant recipients (*n* = 5), or underwent further surgery within 30 days of the previous surgery (*n* = 13), leaving data for 442 patients available for inclusion in the study. One hundred and fifty-one patients underwent pulmonary surgery and 291 underwent non-pulmonary surgery with IP

Retrospective review of the clinical records identified 50,394 patients who underwent surgery under general anesthesia from April 2008 to October 2017. Of these patients, 1789 had IP. In total, 1345 patients were excluded because they had not undergone HRCT (*n* = 422), did not have IP confirmed on HRCT of the chest (*n* = 905), were lung transplant recipients (*n* = 5), or underwent further surgery within 30 days from the previous surgery (*n* = 13), leaving data for 444 patients available for inclusion in the study. One hundred and fifty-one of the patients underwent pulmonary surgery and 293 underwent non-pulmonary surgery (Fig. 1). No patients were treated by antifibrotic agents at the time of surgery. Ninety-nine (65.1%) of the 151 patients in the pulmonary surgery group underwent operations for lung cancer and 53 (34.9%) for other lung conditions. The pulmonary surgery group underwent segmentectomy (39.7%), lobectomy (11.9%), or pneumonectomy (48.3%). The procedures performed in the 291 patients in the non-pulmonary surgery group were intestinal (*n* = 78, including 3 esophagus, 16 stomach, and 59 small intestine and colon), orthopedic (*n* = 74), cardiac (*n* = 44), hepatic, gallbladder, or pancreatic (*n* = 19), otolaryngologic (*n* = 18), breast (*n* = 15), urological (*n* = 12), neurosurgery (n = 9), gynecologic (*n* = 10), or other (*n* = 11). The characteristics of each group are shown in Table 1. The proportions of patients who were male (76.2% vs 59.5%, pulmonary surgery vs non-pulmonary surgery, respectively), had a history of smoking (70.1% vs 65.0%), %DL_CO_ on pulmonary function tests (65.2% vs 51.3%), and tumor surgery (63.6% vs 41.9%) were higher in the pulmonary surgery group than in the non-pulmonary surgery group. In the pulmonary surgery group, 92 patients (61.9%) had pulmonary diseases other than IPF, including 63 (41.7%) with idiopathic interstitial pneumonias other than IPF, 21 (13.9%) with connective tissue disease-related IP, 3 (1.9%) with chronic hypersensitivity pneumonia, and 5 (3.3%) with other conditions. In the non-pulmonary surgery group, 202 patients (69.4%) had pulmonary disease other than IPF, including 99 (34.0%) with idiopathic interstitial pneumonias other than IPF, 93 (32.0%) with connective tissue disease-related IP, 3 (1.0%) with chronic hypersensitivity pneumonia, and 7 (2.4%) other conditions. The JACS risk score was higher in the pulmonary surgery group (9.5) than in the non-pulmonary surgery group (7.0). The patients who underwent pulmonary surgery were younger than those who underwent non-pulmonary surgery (mean age 69 years vs 71 years). Fewer patients in the pulmonary surgery group had received corticosteroid therapy (17.3% vs 29.6%). The serum LDH (193 U/L vs 206 U/L), frequency of emergency surgery (0.0% vs 4.4%), and frequency of neoadjuvant chemotherapy (1.1% vs 9.8%) were lower in the pulmonary surgery group than in the non-pulmonary surgery group. AE-IP developed in 5 (3.3%) of the 151 patients who underwent pulmonary surgery and in 4 (1.4%) of the 291 who underwent non-pulmonary surgery; the difference was not statistically significant (Table 1). Other surgical complications occurred in 18 (11.9%) of the patients in the pulmonary surgery group and in 40 (13.7%) in the non-pulmonary surgery group; the difference was not statistically significant. Lung infections occurred as a surgical complication in 10 patients (6.6%) in the pulmonary surgery group and in 8 patients (2.8%) in the non-pulmonary surgery group; again, the difference was not statistically significant. Other complications associated with the surgical procedure were suture failure, organ fistula and subcutaneous emphysema (*n* = 16; 3 in the pulmonary surgery group and 13 in the non-pulmonary surgery group), arrhythmia (*n* = 4; 1 in the pulmonary surgery group and 3 in the non-pulmonary surgery group), sepsis (*n* = 3; all in the non-pulmonary surgery group), wound infection (*n* = 3; all in the non-pulmonary surgery group) and ileus (*n* = 3; 1 in the pulmonary surgery and 2 in the non-pulmonary surgery group), and others.

**Table 1:** Characteristics of patients who underwent pulmonary surgery or non-pulmonary surgery

Characteristics	Pulmonary surgeries			Non-pulmonary surgeries			*P* value
		% or (IQR)	[*n*]		% or (IQR)	[*n*]	
N, no	151	34.0%		291	66.0%		
Acute exacerbation, no	5	3.3%	[151]	4	1.4%	[291]	*P* = 0.284
Age, median	69	(65.0–78.0)	[151]	71	(63.0–75.0)	[291]	*p* = 0.027†
Sex Male, no	115	76.2%	[151]	173	59.5%	[291]	*p* = 0.001†
BMI (kg/m^2^), median	22.1	(19.7–24.9)	[151]	22.6	(19.8–24.8)	[291]	*p* = 0.503
History of smoking, no	114	78.6%	[145]	158	64.8%	[244]	*p* = 0.004†
IPF or other IP, no	59	39.1%	[151]	89	30.6%	[291]	*p* = 0.089
Corticosteroids therapy, no	26	17.3%	[151]	86	29.6%	[291]	*p* = 0.006†
Past acute exacerbation, no	2	1.3%	[151]	7	2.4%	[291]	*p* = 0.725
HRCT findings							
UIP pattern, no	74	49.0%	[151]	130	44.7%	[291]	*p* = 0.421
Emphysema, no	77	51.0%	[151]	106	36.7%	[291]	*p* = 0.004†
Pulmonary function test							
%VC, median (%)	98.5	(85.1–112.8)	[140]	96.5	(79.9–107.9)	[236]	*p* = 0.089
FEV1/FVC, median (%)	76.7	(68.2–82.6)	[140]	77.8	(71.5–83.7)	[236]	*p* = 0.049†
%DLCO, median (%)	65.2	(53.5–81.5)	[127]	51.3	(41.7–70.1)	[49]	*p* = 0.001†
KL-6, median (U/mL)	487	(311–825.5)	[145]	547	(337–923.5)	[145]	*p* = 0.307
LDH, median (U/L)	193	(170–224)	[150]	206	(179–254.5)	[284]	*p* = 0.008†
CRP, median (mg/dL)	0.21	(0.1–0.6)	[151]	0.25	(0.1–1.2)	[287]	*p* = 0.169
JACS risk score, median	10	(6.8–11)	[138]	7	(4–8)	[133]	*p* < 0.001†
Emergency surgery, no	0	0%	[151]	12	4.1%	[291]	*p* = 0.010†
Tumor surgery, no	96	63.6%	[151]	122	41.9%	[291]	*p* < 0.001†
Neoadjuvant chemotherapy, no	1	1.1%	[94]	12	9.8%	[122]	*p* = 0.008†
Anesthesia time, median (min)	273	(191–336)	[141]	271	(187–388)	[277]	*p* = 0.409
Amount of bleeding, median (mL)	70	(15.0–180.5)	[141]	110	(23.0–450.0)	[275]	*p* = 0.002†
Lung infection	10	6.6%	[151]	8	2.8%	[291]	*p* = 0.073
(surgical complication), no							

*IQR* interquartile range, *BMI* body mass index, *IPF* idiopathic pulmonary fibrosis, *IP* interstitial pneumonia, *HRCT* high resolution computed tomography, *UIP* usual interstitial pneumonia, *VC* vital capacity, *FEV1/FVC* forced expiratory volume in 1 second/forced vital capacity, *DLCO* diffusing capacity for carbon monoxide, *KL-6* Klebs von den Lungen-6, *LDH* lactate dehydrogenase, *CRP* C-reactive protein, *JACS* Japanese association for chest surgery

†; *P* value < 0.05, Fisher’s exact test or Wilcoxon test

### Characteristics of patients in the two study groups who developed AE-IP

In the pulmonary surgery group, more patients who developed AE-IP had received corticosteroid therapy preoperatively (60.0% vs 15.8%) than those who did not; serum CRP levels were also higher in the patients who developed AE-IP than in those who did not (0.54 mg/dL vs 0.21 mg/dL). However, there were no significant differences in any of the variables measured between the patients who developed AE-IP and those who did not in the non-pulmonary surgery group (Table 2). CRP levels in patients who developed AE-IP tended to be higher than those in patients who did not in the non-pulmonary surgery group, but not significantly so (7.10 mg/dL vs 0.24 mg/dL, *P* = 0.068). The details of patients who developed AE-IP is shown in Additional file 1.

**Table 2:**
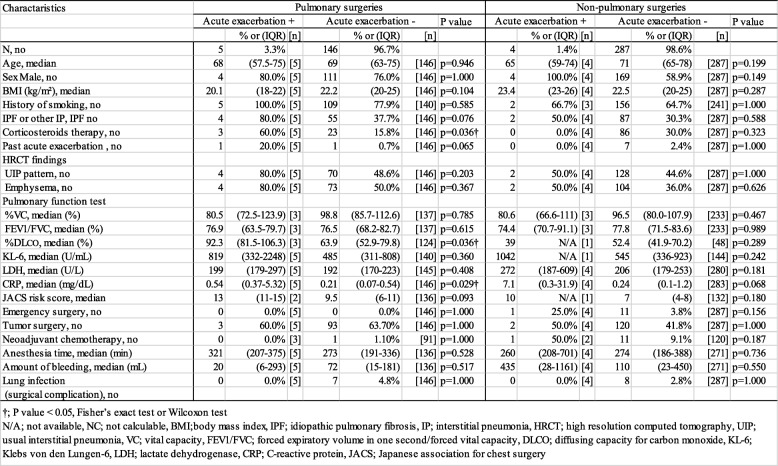
Characteristics of patients who developed acute exacerbation in pulmonary or non-pulmonary surgery

*N/A* not available, *NC* not calculable, *BMI* body mass index, *IPF* idiopathic pulmonary fibrosis, *IP* interstitial pneumonia, *HRCT* high resolution computed tomography, *UIP* usual interstitial pneumonia, *VC* vital capacity, *FEV1/FVC* forced expiratory volume in one second/forced vital capacity, *DLCO* diffusing capacity for carbon monoxide, *KL-6* Klebs von den Lungen-6, *LDH* lactate dehydrogenase, *CRP* C-reactive protein, *JACS* Japanese association for chest surgery

†; *P* value < 0.05, Fisher’s exact test or Wilcoxon test

### Risk factors for AE-IP in pulmonary and non-pulmonary surgery

Table 3 showed the risk factors for acute exacerbation in the pulmonary and non-pulmonary surgery groups. Logistic regression analysis identified corticosteroid therapy (odds ratio 35.75, 95% confidence interval 1.267–1026, *P* = 0.039) and a past history of AE-IP (odds ratio 36.25, 95% confidence interval 1.285–1040, *P* = 0.038) to be significant predictors of AE-IP after pulmonary surgery and serum CRP (odds ratio 1.187, 95% confidence interval 1.073–1.345, *P* = 0.002) to be the only predictor of AE-IP after non-pulmonary surgery.

**Table 3:** Risk factors for acute exacerbation in pulmonary and non-pulmonary surgeries

Charactaristics	Pulmonary surgeries				Non-pulmonary surgeries	
	OR	(95% CI)	*P* value	OR	(95% CI)	*P* value
Age, median	0.997	(0.941–1.094)	*p* = 0.940	0.951	(0.868–1.057)	*p* = 0.332
Sex Male ^−/a^	1.261	(0.179–25.09)	*p* = 0.835	NC	NC	NC
BMI (kg/m^2^)	0.817	(0.601–1.055)	*p* = 0.129	1.114	(0.855–1.438)	*p* = 0.414
History of smoking ^a/−^	1.779	(0.586–20.47)	*p* = 0.224	1.099	(0.103–26.63	*p* = 0.944
IPF or other IP, IPF	6.618	(0.949–131.1)	*p* = 0.569	2.299	(0.272–19.40)	*p* = 0.415
Corticosteroid therapy ^−/a^	35.750	(1.267–1026)	*p* = 0.039†	0.517	(0.044–1.570)	*p* = 0.176
Past acute exacerbation ^−/a^	36.250	(1.285–1040)	*p* = 0.038†	2.039	(0.174–6.686)	*p* = 1.000
HRCT findings						
UIP pattern	4.343	(0.624–86.00)	*p* = 0.146	1.242	(0.147–10.46)	*p* = 0.830
Emphysema	4.000	(0.575–79.20)	*p* = 0.172	1.740	(0.206–14.67)	*p* = 0.585
Pulmonary function test						
%VC	0.985	(0.931–1.039)	*p* = 0.582	0.978	(0.924–1.035)	*p* = 0.445
%VC < 80%	2.115	(0.096–22.89)	*p* = 0.566	1.508	(0.069–16.02)	*p* = 0.745
FEV1/FVC	0.986	(0.894–1.101)	*p* = 0.791	1.014	(0.915–1.157)	*p* = 0.820
%DLCO	1.039	(0.995–1.087)	*p* = 0.081	1.000	(0.997–1.003)	*p* = 0.946
KL-6	1.000	(0.999–1.001)	*p* = 0.469	1.000	(0.997–1.003)	*p* = 0.601
KL-6 > 1000 U/mL ^−/a^	1.150	(0.058–8.193)	*p* = 0.904	3.765	(0.857–45.74)	*p* = 0.113
LDH	1.012	(0.995–1.027)	*p* = 0.153	1.004	(0.999–1.007)	*p* = 0.125
CRP	1.126	(0.844–1.345)	*p* = 0.324	1.187	(1.073–1.344)	*p* = 0.002†
JACS risk score	1.554	(0.983–2.780)	*p* = 0.061	1.363	(0.779–2.301)	*p* = 0.261
Emergency surgery	NC	NC	NC	8.363	(0.396–71.64)	*p* = 0.139
Tumor surgery	0.855	(0.138–6.643)	*p* = 0.867	1.391	(0.165–11.72)	*p* = 0.743
Neoadjuvant chemotherapy ^a/−^	2.936	(0.235–14.01)	*p* = 1.000	9.909	(0.375–262.9)	*p* = 0.144
Anesthesia time	1.002	(0.993–1.009)	*p* = 0.733	1.002	(0.996–1.006)	*p* = 0.406
Amount of bleeding	0.999	(0.992–1.003)	*p* = 0.778	1.000	(0.998–1.000)	*p* = 0.866
Lung infection ^a/a^	1.088	(0.093–3.421)	*p* = 1.000	1.912	(0.164–6.234)	*p* = 1.000
(surgical complication)						

*OR* odds ratio, *CI* confidence interval, *NC* not calculable, *BMI* body mass index, *IPF* idiopathic pulmonary fibrosis, *HRCT* high solution computed tomography, *UIP* usual interstitial pneumonia, *VC* vital capacity, *FEV1/FVC* forced expiratory volume in 1 second/forced vital capacity, *DLCO* diffusing capacity for carbon monoxide, *KL-6* Klebs von den Lungen-6, *LDH* lactate dehydrogenase, *CRP* C-reactive protein, *JACS* Japanese association for chest surgery

†; *P* value < 0.05, Logistic regression analysis, ^a^Penalized Firth correction applied due to quasi-complete data separation

## Discussion

In this study, there was no significant difference in the proportion of patients who developed AE-IP after pulmonary surgery and those who developed AE-IP after non-pulmonary surgery (3.3% vs 1.4%). This is the first retrospective study to compare the frequency of AE-IP after non-pulmonary surgery with that after pulmonary surgery at the same institution.

The literature on AE-IP in patients undergoing non-pulmonary surgery is very limited [15–17]. AE-IP has been reported to occur in 6.3–7.8% of patients with IP after non-pulmonary surgery [16, 17]. However, the frequency of AE-IP after non-pulmonary surgery in the present study was lower than that in the previous studies. Furthermore, AE-IP developed in 5 (3.3%) of 151 patients after pulmonary surgery in our study. This figure is similarly lower than that in previous studies (9.5–22.5%) [6–12] and also lower than in our previous study (8.5%) that was conducted at the same hospital as the present study [20]. We believe that this difference in frequency of AE-IP reflects differences in patient selection. The data for patient age, proportion of men, physical data, laboratory data, and the UIP pattern found on chest HRCT were not markedly different between the studies. However, several reasons for the difference in frequency between our previous study and the present one might be considered. First, the surgical procedures performed in the present study were less invasive than those in our previous study. Segmentectomy, lobectomy, and pneumonectomy were performed in fewer patients in the present study than in the previous one (65.1% vs 76.3%). Another reason for the lower frequency of AE-IP in this study may be that the IP was less severe than that in the previous studies. Our present study included patients with IP who only had a slight reticular shadow on chest HRCT. Moreover, the serum LDH level (median 212 U/L vs 193 U/L) and KL-6 level (median 845.5 U/mL vs 487 U/mL) in the present study were lower and the %DLCO (54.4% vs 65.2%) in the present study was higher than in our previous study. The rate of postoperative AE-IP (3.3%) in the present study was lower than the rates in the studies reported by Maniwa et al. (9.7%) [12] and Sato et al. (9.3%) [8]; furthermore, our rates of male sex (75.7% vs 90.3 and 90.4%, respectively) and surgical procedures other than wedge resection (65.1% vs 98.1 and 84.7%) were lower than those in the two previous studies. Moreover, the median KL-6 level in our study (487 U/mL) was lower than that in the study by Maniwa et al. (759 U/mL). Therefore, we speculate that the IP in our study might have been less severe than that in previous studies in non-pulmonary surgery, given that patients were enrolled in the pulmonary and non-pulmonary surgery groups using the same methods. The annual incidence of adjudicated AE-IPF events was reported to be lower than 5% in the placebo groups included in most of the recent clinical trials [21]. The frequency of AE-IP after pulmonary surgery was lower in the present study than in our previous study, but the frequency of postoperative AE-IP was higher than the frequency of AE-IPF events within 90 days as estimated from the annual incidence of AE-IPF (under 1.23%). However, the rate of postoperative AE-IP was not significantly different between the pulmonary and non-pulmonary surgery groups. Therefore, we should assess patients carefully and explain their risk of an AE-IP whether they are undergoing non-pulmonary or pulmonary surgery. In one study, the mortality rate among patients in whom AE-IP was detected on routine HRCT within a few days postoperatively was reported to be lower than in those who did not have routine HRCT; it was speculated that the most important reason for the lower mortality rate in the group that underwent routine HRCT was that AE-IP was detected early, resulting in earlier initiation of treatment [22]. In this study, only 5 patients in the pulmonary surgery group and 4 in the non-pulmonary surgery group developed AE-IP postoperatively. However, the numbers were small, so it is possible that a difference could be demonstrated between these groups when a large-scale multicenter study is performed in the future. The only potential risk factor for AE-IP after non-pulmonary surgery in this study was the CRP level. A recent study found that a high preoperative CRP level was a risk factor for AE-IP after non-pulmonary surgery [17]. Our study supports the finding of that study. Several previous studies have suggested viral infection as a possible cause of AE-IP [23, 24] and there has been a report indicating that the CRP level is a significant prognostic factor in AE-IP [1]. These findings suggest that inflammation could be one of the pathogenic mechanisms contributing to AE-IP [1, 25]. However, CRP levels have also been reported to be elevated in both infection and AE-IP, but are much higher in infection than in AE-IP [1]. The finding that the serum CRP level is a predictor of AE-IP suggests that acute respiratory distress syndrome (ARDS) may have been triggered by infection in our patients who underwent non-pulmonary surgery. ARDS is a clinical syndrome that is defined as the rapid onset of hypoxia with a partial pressure of oxygen in arterial blood/fraction inspired oxygen ratio of ≤300 and bilateral pulmonary infiltrates in the absence of left atrial hypertension; the overall in-hospital mortality of patients with ARDS is believed to exceed 30% [26, 27]. The typical pathologic feature of AE-IP is diffuse alveolar damage (DAD) [28, 29], which is also the histologic hallmark of ARDS, although this feature can be found on biopsy in only about half of patients meeting the clinical criteria for a diagnosis of ARDS [30]. Clinical signs of ARDS, including HRCT findings, are very similar to those of AE-IP [31]. Therefore, correct diagnosis of AE-IP is difficult in the clinical setting, particularly in terms of ruling out ARDS [31, 32]. The frequency of ARDS after non-cardiothoracic, vascular, and trauma surgery has been reported to be 0.2% [33, 34], which is lower than that of postoperative AE-IP in the patients who underwent non-pulmonary surgery in our study. Corticosteroid therapy and a past history of AE-IP were potential risk factors for AE-IP after pulmonary surgery in the present study and were reported to be one of the risk factors for AE-IP after pulmonary resection in patients with lung cancer [14]. However, corticosteroid therapy and a past history of AE-IP were not risk factors for AE-IP in our non-pulmonary surgery group, which might reflect the small number of cases of AE-IP in the present study or other mechanisms between pulmonary and non-pulmonary AE-IP. A previous study reported that use of propofol was a predictor of AE-IP in patients undergoing non-pulmonary surgery [16]. However, propofol was not a predictive factor in the present study, although almost all of the patients received propofol when they underwent surgery.

This study has several limitations that should be considered when interpreting the results. The first is its retrospective single-center design. There was a lot of missing data, which might have affected the incidence of AE-IP in our study population, especially in the non-pulmonary surgery group. Second, we had very few cases because the frequency of AE-IP after surgery was low. A randomized study containing a sufficient number of patients to allow a statistical analysis is now required to identify the frequency and risk factors for postoperative AE-IP in patients undergoing non-pulmonary surgery.

## Conclusions

This is the first retrospective study to compare the frequency of AE-IP after pulmonary surgery with that after non-pulmonary surgery. Our results suggest that the incidence of postoperative AE-IP in patients undergoing non-pulmonary surgery is slightly lower but not significantly different from that in those undergoing pulmonary surgery. An elevated preoperative CRP level is a possible risk factor for AE-IP after non-pulmonary surgery. A multicenter study is now needed to clarify the preoperative risk factors for AE-IP after non-pulmonary surgery.

## Additional file

Additional file 1: Characteristics of patients with developing acute exacerbation of interstitial pneumonia. (XLSX 11 kb)

## Data Availability

Not applicable.

## Abbreviations

AE-IP: Acute exacerbation of interstitial pneumonia
ARDS: Acute respiratory distress syndrome
CRP: C-reactive protein
CT: Computed tomography
DAD: Diffuse alveolar damage
HRCT: High resolution computed tomography
IP: Interstitial pneumonia
IPF: Idiopathic pulmonary fibrosis
IQR: Interquartile range
JACS: Japanese Association for Chest Surgery
KL-6: Klebs von den Lungen-6
OR: Odds ratio
UIP: Usual interstitial pneumonia
